# The Content of Tocols in South African Wheat; Impact on Nutritional Benefits

**DOI:** 10.3390/foods6110095

**Published:** 2017-11-02

**Authors:** Maryke Labuschagne, Nomcebo Mkhatywa, Eva Johansson, Barend Wentzel, Angeline van Biljon

**Affiliations:** 1 Department of Plant Sciences, University of the Free State, Bloemfontein 9300, South Africa; LabuscM@ufs.ac.za (M.L.); 2006041384@ufs4life.ac.za (N.M.); avbiljon@ufs.ac.za (A.v.B.); 2 Department of Plant Breeding, The Swedish University of Agricultural Sciences, Box 101, SE-230 53 Alnarp, Sweden; 3 Small Grains Institute, Bethlehem 9700, South Africa; wentzelb@arc.agric.za

**Keywords:** *Triticum aestivum*, tocopherol, tocotrienol, vitamin E, genotype, environment

## Abstract

Wheat is a major component within human consumption, and due to the large intake of wheat, it has an impact on human nutritional health. This study aimed at an increased understanding of how the content and composition of tocols may be governed for increased nutritional benefit of wheat consumption. Therefore, ten South African wheat cultivars from three locations were fractionated into white and whole flour, the content and concentration of tocols were evaluated by high performance liquid chromatography (HPLC), and vitamin E activity was determined. The content and composition of tocols and vitamin E activity differed with fractionation, genotype, environment, and their interaction. The highest tocol content (59.8 mg kg^−1^) was obtained in whole flour for the cultivar Elands grown in Ladybrand, while whole Caledon flour from Clarence resulted in the highest vitamin E activity (16.3 mg kg^−1^). The lowest vitamin E activity (1.9 mg kg^−1^) was found in the cultivar C1PAN3118 from Ladybrand. High values of tocotrienols were obtained in whole flour of the cultivars Caledon (30.5 mg kg^−1^ in Clarens), Elands (35.5 mg kg^−1^ in Ladybrand), and Limpopo (33.7 mg kg^−1^ in Bultfontein). The highest tocotrienol to tocopherol ratio was found in white flour (2.83) due to higher reduction of tocotrienols than of tocopherols at fractionation. The quantity and composition of tocols can be governed in wheat flour, primarily by the selection of fractionation method at flour production, but also complemented by selection of genetic material and the growing environment.

## 1. Introduction

Wheat is, together with rice, the major food crop in the world, supporting 20% of the daily energy for the human population [1]. In certain parts of the world, wheat is even the main staple food, contributing up to 70% of the daily energy and protein in the human diet [2]. Due to its high consumption, wheat provides a significant amount of energy, proteins, and selected micronutrients and vitamins to the consumer [3,4,5]. Thus, despite the fact that food from other origins might to a large extent have a higher relative content of certain compounds, wheat serves as a source of important nutritional components such as iron and zink, vitamin E, phenolics, and carotenoids [6]. The content of vitamin E and its activity is determined by the content of certain tocols [7]. The tocols are known as bioactive compounds with antioxidant traits, for e.g., they prevent oxidation of double bonds by reacting with peroxyl radicals and protecting lipids and membrane proteins against oxidative stress [8,9]. Tocols cannot be produced by humans and are, therefore, important components of any diet, and they are obtained from a large number of plant based foods [6]. Tocols consists of eight lipid-soluble compounds: α-, β-, γ-, δ-tocotrienol, and α-, β-, γ-, δ-tocopherol. Previous literature has accounted different vitamin E activity to the various tocopherols due to their chemical structures and physiological factors. However, recent opinion considers only α-tocopherol as the source of vitamin E activity [7]. For whole wheat flour, a mean vitamin E content of 0.71 mg/100 g has been reported by the U.S. Department of Agriculture (USDA) [10]. However, higher levels of vitamin E have been reported in whole wheat flour from certain genetic material, resulting in the presence of 20% of the daily requirement of vitamin E in 200 g of whole wheat flour [11]. Poor nutritional status together with a high prevalence of stressors, for e.g., malaria and HIV as may be the status of many in developing countries, are known to contribute towards vitamin E deficiency [12]. Thus, selection of suitable wheat material with high vitamin E content might contribute to solving the problems of vitamin E deficiency. Tocotrienols do not show vitamin E activity but are known to have higher antioxidant activity than the tocopherols and also have additional important health promoting effects [11,13]. Tocopherols are widely distributed in higher plants whereas tocotrienols occur mainly in some non-photosynthetic tissues such as seeds and endosperm of monocot grains [14]. In the wheat grain, α- and β-tocopherols are mainly found in the wheat germ, while tocotrienols are concentrated in the pericarp, testa, aleurone, and in the endosperm. 

Fractionation of the crushed grain during milling is known to have a critical implication for the distribution of many nutrients [15]. The consumption of whole grain is a healthy alternative to white flour [16]. Previous studies have also shown genotype, environment, and cultivation practices to have an impact on tocol content and composition [6,11,14]. Increased understanding of the impact and interactions of genotype, environment, and processing on content of tocols and vitamin E activity in wheat flour will positively impact a healthy intake of these compounds from wheat. 

Thus, the aim of this study was to evaluate the effects of genotype, environment, and fractionation through milling on the quantity and composition of tocol components in South African wheat flour. Interactions as well as importance of the various evaluated factors will be investigated and conclusions drawn as related to opportunities to govern these health components in wheat flour. 

## 2. Materials and Methods

### 2.1. Plant Material

Ten South African bread wheat cultivars, Betta-DN, Caledon, Elands, Gariep, Komati, Limpopo, Matlabas, PAN3118, PAN3349, and PAN3377, grown in four replicates in each location, were used in the present study. The trials were conducted at three different locations in one season: Bultfontein (28°16′53.14″ S 26°27′02.77″ E, north western Free State with low rainfall, high temperatures, high evaporation requirements and deep, yellow sandy loam soils with a water table present), Ladybrand (29°14′30.75″ S 27°20′18.55″ E, central Free State, moderate rainfall, moderate temperatures, a lower evaporation requirement and relatively shallow duplex soils), and Clarens (28°24′26.63″ S 27°20′18.55″ E, eastern Free State, higher rainfall, lower temperatures, lower evaporation requirement with predominantly yellow soils of average effective depth). The trials were planted under dryland conditions in a randomized complete block design. Trial plots consisted of five rows of 5 m length and inter-row spacing of 5 cm. Fertilization was done after soil analysis and according to normal production practices for each location. 

### 2.2. Extraction of Tocols

Milled grain samples were freeze dried for three days before tocol extraction. The extraction [17] was performed with modifications as suggested by Labuschagne et al. [18]. 

### 2.3. Analytical High Performance Liquid Chromatography (HPLC)

**Figure 1:**
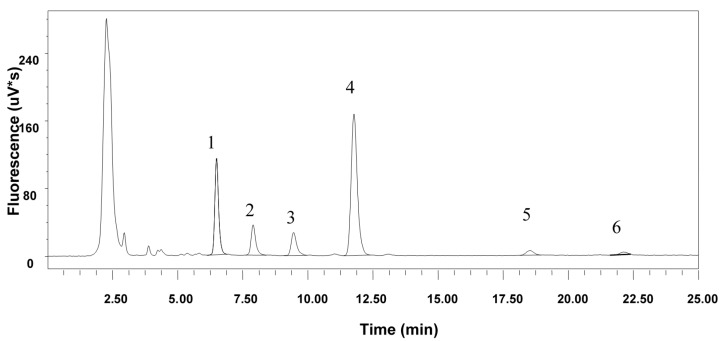
Example of separation of tocols by HPLC (High Performance Liquid Chromatography) from one wheat sample. Peak 1 = α-tocopherol, Peak 2 = α-tocotrienol, Peak 3 = β-tocopherol, Peak 4 = β-tocotrienol, Peak 5 = δ-tocopherol, and Peak 6 = δ-tocotrienol.

A normal phase-HPLC method [19] with modification [18] was used to separate the tocol compounds (Figure 1). A Phenomenex Luna Silica column (250 mm × 4.6 mm inner diameter (i.d.), 5 μm particle size) was used. The mobile phase was *n*-hexane/ethyl acetate/acetic acid (97.3:1.8:0.9 *v*/*v*/*v*) at a flow rate of 1.6 mL min^−1^. All peaks were detected by fluorescence and the wavelength of detection was set to 290 nm and emission wavelength of 330 nm. HPLC injection volume was 10 μL per injection. A standard solution was used to carry out the linearity test over the different concentration ranges (ng μL^−1^) close to the amount of tocols found in the samples: α-tocopherol 0.47–9.57 ng μL^−1^; β-tocopherol 0.23–4.7 ng μL^−1^; γ-tocopherol 0.65–13.1 ng μL^−1^; δ-tocopherol 0.62–12.4 ng μL^−1^; β-tocotrienol 0.54–10.82 ng μL^−1^. Total tocols were the sum of α-tocopherol, β-tocopherol, α-tocotrienol, β-tocotrienol, and δ-tocotrienol. 

### 2.4. Data Analysis

Statistical evaluation applying ANOVA followed by mean comparison with Duncan post-hoc test at *p* < 0.05, Spearman rank correlation analyses, and Principal component analyses (PCA) was carried out using the statistical package SAS (2004; SAS Institute Inc., Cary, NC, USA). In order to explain the proportion of the contribution of variation by the environments, genotypes, and flour fractionation on the tocol composition, regression analysis was applied [20,21]. Vitamin E activity was calculated based on α-tocopherol content according to the Scientific Opinion of an EFSA (European Food Safety Authority) Panel [7]. The Recommended Daily Intake (RDI) of vitamin E set by European Parliament and the Council in the Regulation No. 1169/2011 of 25 October 2011 is 12 mg/day [22]. 

## 3. Results

### 3.1. Importance of Genotype, Environment, and Fractionation on Tocols Content and Composition

**Figure 2:**
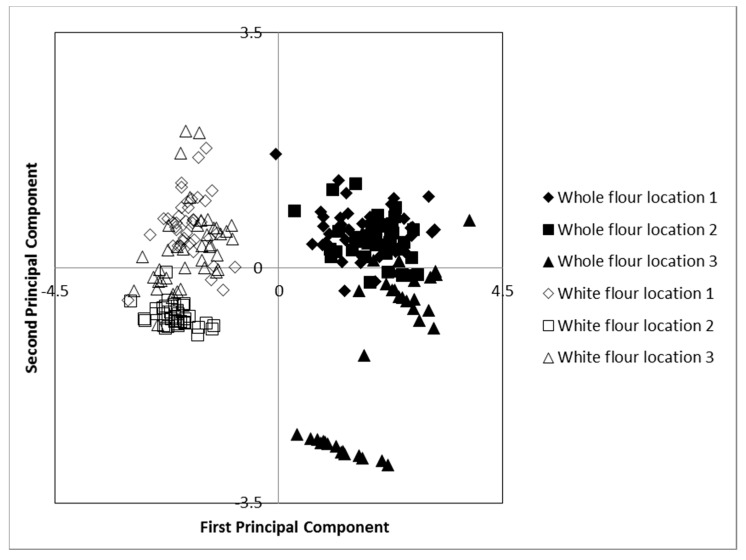
Loading plot from principal component analysis of tocopherols in two flour types of ten varieties grown at three locations in South Africa. Location 1 = Bultfontein, Location 2 = Clarens, Location 3 = Ladybrand. First principal component explained 79.3% of the variation while the second principal component explained 17.4% of the variation.

The percentage recovery of tocols was more than 95%, and the different tocols were successfully separated by HPLC (Figure 1). The major tocols found were α- and β-tocopherol and α- and β-tocotrienol (Figure 1). Delta-tocopherols and especially γ-tocopherols were only found in small amounts and often only in traces. Flour type (white flour versus whole flour) was shown to explain by far the highest part of the variation in tocols content and composition except for δ-tocotrienol, where location was a significant parameter for the variation (Table 1). However, combination of flour type, cultivar, and location resulted in a higher degree of explanation as compared to each of the factors alone (Table 1), and analysis of variance (ANOVA) also showed significant interactions among the factors for content and composition of the tocols (*p* < 0.01). Similarly, the PCA analysis showed samples clearly clustering in two separate groups as related to flour type, although a number of whole flour samples from Ladybrand were also diverging into a separate group of whole flour samples based on lower values on the second principal component value (Figure 2).

### 3.2. Effect of Flour Type on Content and Composition of Tocols

Whole flour had significantly (*p* < 0.005) higher tocol concentrations than white flour, the latter having on average 40% of the concentration of whole flour (Table 2). Concentration in white flour of α-tocopherol was 24% of that in whole flour and concentration in white flour of β-tocopherol was 31% of that in whole flour. Furthermore, white flour contained 25% of the α-tocotrienol concentration found in whole flour and 53% of the β-tocotrienols. The fact that the β-tocotrienols were retained to a higher degree than the other tocols from the whole to the white flour could also be seen as a higher tocotrienol to tocopherol quota (TT/TP) in the white flour as related to whole flour (Table 2). Tocol concentration in white flour as a percentage of that in whole flour varied in genotypes and locations, from 31% (PAN 3349 in Ladybrand) to 51% (Caledon at Bultfontein; Table 3).

### 3.3. Effect of Cultivar and Growing Location on Content and Composition of Tocols

Tocol concentration in genotypes ranged between 16.49–25.49 mg kg^−1^ for white flour and 41.92–54.87 mg kg^−1^ for whole flour (Table 3). Thus, significant differences in tocol content and composition were found among certain of the evaluated cultivars (Table 3), despite the fact that only a limited amount of variation was explained by the differentiation in cultivars (Table 1 and Figure 2). Among the evaluated cultivars, Caledon was found to have a high concentration of tocols in whole flour and the highest concentration among the cultivars in white flour.

Cultivation of the cultivars in Ladybrand resulted in a higher tocol concentration in whole flour and a higher concentration in the white flour as compared to that of the other localities, with an average of 20.1 mg kg^−1^ for white flour and 52.3 mg kg^−1^ for whole flour (Table 3). In general, total tocol content in the samples differed significantly when grown at the different localities for whole flour but no such significant differences were found for white flour. Part of the explanation for this variation in the whole flour (Figure 2) might be the presence of δ-tocotrienol in samples grown in Ladybrand which were only found in trace amounts in samples from the other growing locations. However, a significant variation was found for the various tocol compound concentrations in both white and whole flour from the different locations. Samples from Clarens and Ladybrand had a significantly higher concentration of α-tocopherol in white and whole flour and β-tocopherol in white flour as compared to samples from Bultfontein, while samples from Clarence were higher than those from the other localities for β-tocopherol in whole flour. Significantly higher concentrations of α-tocotrienol and β-tocotrienol were found in whole flour samples from Ladybrand as compared to samples from the other locations, while for white flour, samples from Ladybrand and Bultfontein showed higher concentrations of α-tocotrienol than samples from Clarence (Table 4). In general, samples in Ladybrand showed a relatively high content of both tocopherols and tocotrienols in both white and whole flour, while samples from Clarence showed a relatively high tocotrienol concentration in both types of flours. Samples from Bultfontein showed a relatively high content of tocotrienols only in white flour. Due to differences in variations of concentrations of various tocol compounds by location, the tocotrienol to tocopherol quota (TT/TP) of the samples differed among locations; with significantly higher values in both white and whole flour samples from Ladybrand and Bultfontein as compared to those from Clarence (Table 4). 

## 4. Discussion

The present study clearly shows that the quantity and composition of tocols can be governed in South African wheat flour; fractionation through milling is the major determinant, but the effect was found to interact with the selection of genetic material and the growing environment. As can be seen from calculations of daily requirements of vitamin E, only 5% on average was obtained from white South African wheat flour while 22% was obtained from the corresponding whole flour (Table 5). However, by selecting whole flour of a high vitamin E cultivar (Caledon) grown on the locality (Clarence) contributing most to a high level of vitamin E activity, 27% of the daily requirement could be obtained by consumption of 200 g wheat flour per day (Table 5). Tocols are known to be destroyed when heated (25–94% reduction in vitamin E activity) [23,24]. A recent investigation showed a 40% reduction in tocopherols in bread as compared to their corresponding flour, although toasting of the bread resulted in an increase in tocopherol content so that the content reached 89% of the original content of the flour [25]. Today, most wheat is consumed after a heat treatment, thereby reducing the tocol content; consumption of wheat as whole and/or sprouted grain products is the best solution for wheat to be a tocol source [11]. However, the findings that toasting increases the amount of tocopherols as related to what is found in the bread calls for additional evaluations of how to best process wheat products in order to use these products as tocol sources [25]. To secure a high intake of tocols from the food, flour based products should be combined with other food items very high in tocols content [6,26,27]. 

Vitamin E activity is primarily based on the presence of tocopherols in the food [24,28,29,30]. Besides variation in vitamin E activity, a large variation was also noted in the quantity and composition of tocotrienols in the present wheat material, and this related to variation in fractionation through milling, genotype, and location. Several investigations and results have indicated that tocotrienols are potentially as important for human health as are tocopherols. The tocotrienols have been found to have higher antioxidant capacity [31] and different health promoting properties than those of tocopherols [32,33,34,35]. Examples of biological tocotrienols-mediated activities not shared with the tocopherols are neuroprotection, radio-protection, anti-cancer, anti-inflammatory, and liquid lowering properties [36,37,38,39]. Similar to what was found for vitamin E activity, fractionation was by far the most important factor for the quantity of the tocotrienols found in the flour. However, tocotrienols content decreased less than tocols content with milling to white flour, resulting in a higher tocotrienols to tocols content in the white flour as compared to that of whole flour. Also, cultivation location resulting in the highest tocotrienols content varied for tocotrienols as compared to vitamin E activity. For tocotrienols, Ladybrand resulted in the highest amount among the locations with Caledon, Limpopo, and Elands showing the highest values among the cultivars. Scientifically based recommendations as to daily intake are only available for vitamin E [11] and not for the separate isoforms, although retailers are announcing that 34–43 mg per day of tocotrienols are beneficial [40]. If such a level should be recommended, the present white and whole flour samples would contribute to around 6%and 12%, respectively, of the daily requirements at a consumption of 200 g of flour per day. However, recent literature reports highly divergent bioavailability of tocotrienols based on the source of tocotrienols and on the target population [36]. Various sources of tocotrienols are rich to different degrees in different tocotrienol compounds, for e.g., tocotrienols from palm oil (the most common source of tocotrienol supplements) is particularly rich in δ-tocotrienol, while β-tocotrienol was the predominant compound in the present material, as also shown in previous studies on wheat [41,42]. The fact that the composition of tocotrienols varies with the source of tocotrienols may be one explanation for the difficulties of making recommendations for the daily intake requirements. Also, it is unclear what effect the relationship of the various tocotrienols and the ratio between tocotrienols and tocopherols is playing. Similar to previous studies [43], we were able in this study to show that cultivar and cultivation location affect composition of tocotrienols, for e.g., cultivation in Ladybrand did not only result in high levels of tocotrienols but also in higher levels of δ-tocotrienol in the whole flour than cultivation in the other locations. Ladybrand has a moderate rainfall and temperatures, resulting in lower evaporation and a relatively shallow duplex soil, growing parameters that might be the background for the content and composition of tocols. However, relationships among growing parameters and tocols content and composition have to be further evaluated before conclusions are made. 

Scientific literature recommends not setting daily requirements of tocotrienols until more research based evidence is available [44]. However, from our study, we can conclude that fractionation, cultivation place, and cultivar need to be taken into consideration together with processing of the food in order to determine health effects of a certain food source of tocotrienols. 

An increased understanding of how various tocol compounds can be governed through various production factors, including fractionation, genotype, and locality, opens up opportunities of producing specifically useful raw material for further food processing into nutritional beneficial food products with high bioavailability of wanted compounds. Fractionation was the factor effecting quantity of all tocols to the highest extent. In general, grain antioxidants are known to be concentrated in the bran and germ fractions, and thus these fractions are the major contributors to the total antioxidant activities of wheat [45,46]. A number of studies have also shown significantly more antioxidant activity in products manufactured with whole grains than products from refined wheat [47,48]. Previous studies have indicated that tocopherols are more concentrated in the germ fraction, while tocotrienols are found more in bran and are also observed in the endosperm [16,49]. This agrees with the findings in this study, which showed a higher content of all tocols in the whole flour compared to the white flour, but with the reduction to different extents depending on the compound. Fractionation procedures might, therefore, be a useful tool to effectively modulate tocol content and composition in wheat flour, although it can be complimented with genetic impact through the choice of cultivars and environmental impact through the choice of cultivation location. 

## 5. Conclusions

The quantity and composition of tocols can be governed in wheat flour, primarily by the selection of the fractionation method at flour production, but also complemented by selection of genetic material and the growing environment. Total high content of tocopherols in the flour can best be obtained by the selection of whole flour from a high tocol producing cultivar cultivated in good conditions (not hot and dry). By doing so, 27% of the human daily requirement of vitamin E can be received by an average consumption of 200 g of wheat flour per day unless the tocols in the wheat flour are destroyed by harsh processing conditions.

Tocotrienols are possibly even more important than vitamin E in their contribution from wheat towards a healthy diet. Content and composition of these compounds can also be governed in wheat, as large variations are present due to fractionation, cultivar, and cultivation location. However, to do so, an increased knowledge on the requirements as to total amount, composition, and ratios of the different compounds is needed. 

## Figures and Tables

**Table 1:** Percentage of explanation (obtained through R-square from simple linear regression) of flour types (Flour; F), varieties (V), and growing locations (L) as well as their combinations on various tocols.

	α-TP	β-TP	α-TT	β-TT	δ-TT	TP	TT	Tot
Flour	89.0	89.6	87.8	83.5	11.2	89.9	85.8	90.3
Variety	1.06	1.45	3.53	1.46	4.29	1.18	2.06	1.64
Location	0.90	0.01	0.46	1.38	15.1	0.49	1.16	0.70
F, V, L	90.9	91.1	91.8	86.4	30.7	91.6	89.0	92.6

TP = tocopherols, TT = tocotrienols, Tot = total tocols.

**Table 2:** Mean values (mg kg^−1^) of various tocols depending on flour type (white versus whole meal flour).

Flour	α-TP	β-TP	α-TT	β-TT	δ-TT	TT/TP	Tot
White	3.34 ^b^	2.00 ^b^	1.24 ^b^	12.7 ^b^	0.45 ^b^	2.83 ^a^	19.7 ^b^
Whole	13.3 ^a^	6.43 ^a^	4.93 ^a^	24.0 ^a^	0.60 ^a^	1.49 ^b^	49.4 ^a^

Average values followed by the same letters are not significantly different at *p* < 0.05 applying Duncan post-hoc test. TP = tocopherols, TT = tocotrienols, Tot = total tocols.

**Table 3:** Total tocol content (mg kg^−1^) mean values of ten cultivars in three locations in white and whole flour.

Flour Type	Cultivar	Bultfontein	Clarens	Ladybrand	Average
White	Betta-DN	21.2	17.5	19.0	19.3 ^cd^
	Caledon	26.4	24.1	25.9	25.5 ^a^
	Elands	16.4	20.6	23.8	20.3 ^bc^
	Gariep	20.1	20.0	24.7	21.6 ^b^
	Komati	20.6	21.4	23.6	21.9 ^b^
	Limpopo	17.9	20.7	17.4	18.6 ^cde^
	Matlabas	18.8	18.7	18.3	18.6 ^cde^
	C1PAN3118	17.1	17.0	15.3	16.5 ^f^
	C2PAN3349	18.0	16.6	15.9	16.8 ^f^
	C3PAN3377	18.2	17.8	17.8	18.1 ^def^
	Average	19.5 ^a^	19.4 ^a^	20.2 ^a^	
Whole	Betta-DN	48.0	51.2	52.8	50.6 ^bc^
	Caledon	52.0	55.0	57.6	55.0 ^a^
	Elands	49.9	51.4	59.8	53.7 ^a^
	Gariep	40.9	48.6	49.6	46.3 ^d^
	Komati	43.8	51.8	52.3	49.3 ^bc^
	Limpopo	55.7	49.5	55.7	53.7 ^a^
	Matlabas	39.4	52.8	52.0	48.2 ^cd^
	C1PAN3118	43.5	42.9	45.4	43.9 ^e^
	C2PAN3349	40.4	41.7	43.6	41.9 ^e^
	C3PAN3377	50.2	47.7	54.5	51.0 ^b^
	Average	46.5 ^c^	49.2 ^b^	52.3 ^a^	

Average values followed by the same letters are not significantly different at *p* < 0.05 applying Duncan post-hoc test.

**Table 4:** Average content of each tocol compound (mg kg^−1^) in three locations from white and whole wheat.

Flour Type	Characteristic	Bultfontein	Clarens	Ladybrand
White	α-Tocopherol	3.02 ^b^	3.59 ^a^	3.36 ^a^
	β-Tocopherol	1.88 ^b^	2.08 ^a^	2.02 ^a^
	α-Tocotrienol	1.32 ^a^	1.12 ^b^	1.28 ^a^
	β-Tocotrienol	12.7 ^a^	12.4 ^a^	13.0 ^a^
	TT/TP	3.07 ^a^	2.51 ^b^	2.92 ^a^
Whole	α-Tocopherol	11.8 ^b^	14.1 ^a^	14.0 ^a^
	β-Tocopherol	6.30 ^b^	6.62 ^a^	6.33 ^b^
	α-Tocotrienol	4.80 ^b^	4.45 ^c^	5.52 ^a^
	β-Tocotrienol	22.7 ^c^	23.4 ^b^	26.0 ^a^
	TT/TP	1.53 ^a^	1.36 ^b^	1.57 ^a^

TT = Tocotrienol, TP = Tocopherols; Average values of the different characteristics followed by the same letters are not significantly different at *p* < 0.05 applying Duncan post-hoc test.

**Table 5:** Vitamin E activity of wheat flour of different fractions, genotypes, and localities, calculated as tocopherol equivalents [7] and percentage of recommended intake [11,19] from the average flour consumption in the world of 200 g/person/day.

Flour Origin	Vitamin E Activity (mg kg^−1^)	Recommended Daily Intake (mg)	Percentage of Recommended Vitamin E from 200 g of Wheat Flour (%)
White	3.3	12	5.5
Whole	13.3	12	22.2
*Whole flour at various locations*			
Bultfontein	11.9	12	19.8
Clarence	14.1	12	23.5
*Whole flour from Clarence in various cultivars*			
Caledon	16.1	12	26.8
C1PAN3118	12.3	12	20.5
